# Spin current distribution in antiferromagnetic zigzag graphene nanoribbons under transverse electric fields

**DOI:** 10.1038/s41598-021-96636-6

**Published:** 2021-08-24

**Authors:** Jie Zhang, Eric P. Fahrenthold

**Affiliations:** Department of Mechanical Engineering, University of Texas at Austin, Austin, TX 78712 USA

**Keywords:** Electronic properties and devices, Chemical physics

## Abstract

The spin current transmission properties of narrow zigzag graphene nanoribbons (zGNRs) have been the focus of much computational research, investigating the potential application of zGNRs in spintronic devices. Doping, fuctionalization, edge modification, and external electric fields have been studied as methods for spin current control, and the performance of zGNRs initialized in both ferromagnetic and antiferromagnetic spin states has been modeled. Recent work has shown that precise fabrication of narrow zGNRs is possible, and has addressed long debated questions on their magnetic order and stability. This work has revived interest in the application of antiferromagnetic zGNR configurations in spintronics. A general ab initio analysis of narrow antiferromagnetic zGNR performance under a combination of bias voltage and transverse electric field loading shows that their current transmission characteristics differ sharply from those of their ferromagnetic counterparts. At relatively modest field strengths, both majority and minority spin currents react strongly to the applied field. Analysis of band gaps and current transmission pathways explains the presence of negative differential resistance effects and the development of spatially periodic electron transport structures in these nanoribbons.

## Introduction

Zigzag graphene nanoribbons (zGNRs) have attracted considerable research interest as potential components of spintronic devices, including logic gates ^1^, spin filters ^2^, and field effect transistors ^3^. It is the possibility of forming half-metallic states which has made zGNRs promising candidates for spintronic applications^4,5^. Published work has investigated a variety of techniques aimed at realizing half-metallicity, including doping^5,6^, edge geometry modification^7,8^, and functionalization ^9,10^. The most promising technique for tailoring zGNR performance appears to be the application of external electric fields ^11,12^.

The electronic properties of zGNRs depend in general on their width (n-zGNR denotes a zGNR with a width of ‘n’ carbon chains^5^) and their edge termination. These nanoribbons may be further distinguished by their edge states, which may be ferromagnetic (edge electron spins parallel) or antiferromagnetic (edge electron spins antiparallel). Given the historically difficult^13^ tasks of fabricating narrow nanoribbons with uniform termination, and subsequently measuring their electronic properties, research on the spintronic application of zGNRs has been marked by considerable uncertainty. Initial research enthusiasm for zGNR based spintronics^4,5,14–16^ was tempered by concerns regarding the magnetic order and energetic stability of narrow nanoribbons, in addition to the need for improved fabrication techniques. However the gradual development of new synthesis methods^13,17–19^ has established that bottom-up approaches^13^ can prepare, with atomic precision, very narrow zGNRs. Similarly, recent theoretical and experimental^20,21^ research indicates that the energetically favored magnetic order for zGNRs transitions from ferromagnetic to antiferromagnetic as the nanoribbon width is reduced. As a result, spin current transmission in narrow, undoped, defect-free zGNRs continues to be of considerable spintronics research interest.

The aforementioned device design research is predicated on a thorough understanding of the current transmission physics. Current transmission in zGNRs can be a highly nonlinear function of the applied bias and external fields, since edge states ^5^, band gaps ^14^, ground states ^22^, and other properties may be affected by the applied electrical loads. Although considerable previous work has studied the I–V characteristics of zGNRs as a function of: (a) bias voltage ^23^, and (b) a combination of bias and gate voltages ^24^, research on zGNR performance under the combination of a bias voltage and a transverse electric field has been much more limited. Previous work^14,25–27^ has established the feasibility of using in combination a bias voltage and a transverse electric field for reversible current control ^14,26,28^, and the cited literature includes current-voltage characteristics for transverse field loaded zGNRs in a few configurations, including zGNRs initialized in both ferromagnetic and antiferromagnetic states.

Despite more than a decade of research into the half-metallic zGNR concept, the current transmission performance of even-edge-number zGNRs initialized in an antiferromagnetic spin state remains a matter of debate. This paper presents the first comprehensive investigation of spin current transmission in even-edge-number zGNRs initialized with antiferromagnetic spin (henceforth referred to, as is customary in the related literature, as ‘antiferromagnetic’ zGNRs). The sections which follow show that under the combined loading of a bias voltage and a transverse electric field, both the majority and minority spin currents in antiferromagnetic zGNRs diverge sharply from those predicted for their ferromagnetic counterparts. The spin up current shows a non-monotonic variation (negative differential resistance) with bias voltage, as observed in some deformed zGNR ^29^ and bridged zGNR ^30^ systems. Analysis of the associated band gaps^31^, transmission spectra^32^, charge transfer plots^33^, and transmission pathways may be used to relate the spin current variations to changes in the zGNR electronic structure. Note that unlike previous work presenting transmission pathway plots at a fixed energy, the energy averaged transmission metric introduced here visualizes bulk current flow in the modeled system. The ab initio modeling results presented here may assist in the design of graphene based spintronic devices, since electric field control of zGNR performance may offer simplicity, adaptability, and precision advantages over alternative methods (doping, functionalization, edge modification, zGNR deformation, etc.). In addition, the results presented here indicate that electric field control allows for the adaptive selection of zGNR conductor configurations which emphasize the transmission of total current, up or down spin current, or spin current difference in a single transmission line.

## Computational model

**Figure 1:**
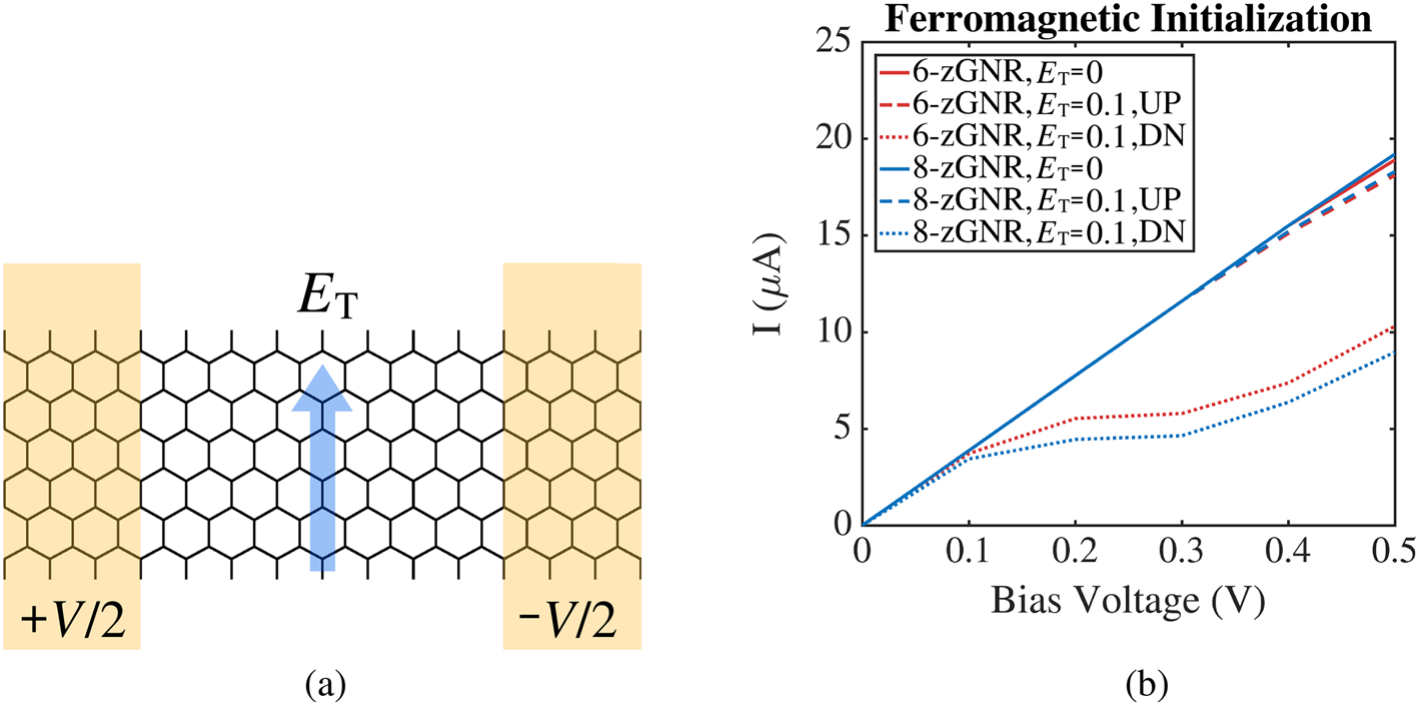
Schematic diagram of a 6-zGNR subjected to a bias voltage and a transverse electric field (left). Total current (solid lines) versus bias voltage for 6 and 8-zGNR at zero transverse electric field strength and spin currents (dotted lines) versus bias voltage for 6-zGNR and 8-zGNR at a transverse electric field strength of $0.1\,{\mathrm {V/}}\AA$ (right).

The physical systems modeled in this paper are zigzag graphene nanoribbons with hydrogen termination, as shown in Fig. 1a. All of the modeled nanoribbons were 14 unit cells in length (for zGNRs the unit cell length is ${\mathrm {2.47\,}}\AA$). A bias voltage of magnitude *V* is applied to the zGNR in the longitudinal direction (the electrodes are shown in yellow) while an electric field of magnitude $E_{\mathrm {T}}$ (indicated by the blue arrow) is applied in the transverse direction. The analysis considers two different zGNR widths, a bias voltage range of 0.0 to 0.9 volts (spanning the values considered in the bulk of the published literature), and transverse electric fields ranging from 0.0 to 0.1 volts per angstrom. Although transverse field strengths an order of magnitude larger have been modeled in some previous work ^9,10^, the modest field strength range considered here is sufficient to achieve significant spin current control effects.

The equilibrium and transport calculations described in this paper were performed using the ab initio code suite SIESTA ^34^. Post-processing and visualization work employed the Python library SISL ^35^ and user-developed codes. The equilibrium calculations employed a local density approximation (LDA) ^36^ exchange correlation functional; the system was relaxed until a maximum atomic force of 0.01 ${\mathrm {eV/}}\AA$ was reached. The analysis employed spin-polarized calculations, using a double-zeta polarized (DZP) basis set for all of the atoms and a mesh cutoff energy of 300 Ry. The transmission calculations employed a general gradient approximation (GGA) with a Perdew-Burke-Ernzerhof (PBE) ^37^ exchange-correlation functional. The Brilloiun zones were sampled with equilibrium $k-$point sets of 3 × 3 × 10; for the transport calculations the $k-$point sets were 3 × 3 × 10 for the scattering region and 3 × 3 × 20 for the electrodes. Pseudopotentials were taken from the National Nanotechnology Infrastructure Network (NNIN) data base ^38^. A separation distance of 20 Åwas maintained between the scattering zone nuclei and the supercell boundaries in the non-transport directions, to preclude supercell interaction effects.

The nanoribbon currents were calculated, for fixed nuclei, using the TranSIESTA module of the SIESTA code suite, which employs a non-equilibrium Green’s function method^39,40^ to model electron transport and a default electronic temperature of 300K. The Landauer-Büttiker formula^41,42^ is used to compute the current

1 $$\begin{aligned} I(V)=\frac{e}{h}\displaystyle \sum _{\sigma }\int _{-\infty }^{+\infty } T_\sigma (E)[f(E-\mu _{\mathrm {R}})-f(E-\mu _{\mathrm {L}})]\,{\mathrm {d}}E \end{aligned}$$

where *V* is the bias voltage, $T_\sigma (E)$ is the transmission coefficient for spin component $\sigma$ at the energy level *E*, *f* is the Fermi function, *e* is the charge on an electron, and *h* is Planck’s constant. The parameters $\mu _{\mathrm {L}}$ and $\mu _{\mathrm {R}}$ are the chemical potentials of the left and right electrodes

2 $$\begin{aligned} \mu _{\mathrm {L}}=E_{\mathrm {F}}-\frac{1}{2}eV ,  \mu _{\mathrm {R}}=E_{\mathrm {F}}+\frac{1}{2}eV \end{aligned}$$

Figure 1b shows a representative set of analysis results, and plots spin current versus bias voltage for 6-zGNR and 8-zGNR at two different transverse electric field strengths. In order to draw contrasts (in later sections) between the performance of ferromagnetic and antiferromagnetic zGNRs, the data shown in this plot is for zGNRs initialized in a ferromagnetic state. All of the remaining results in this paper are computed for zGNRs initialized in an antiferromagnetic configuration.

**Figure 2:**
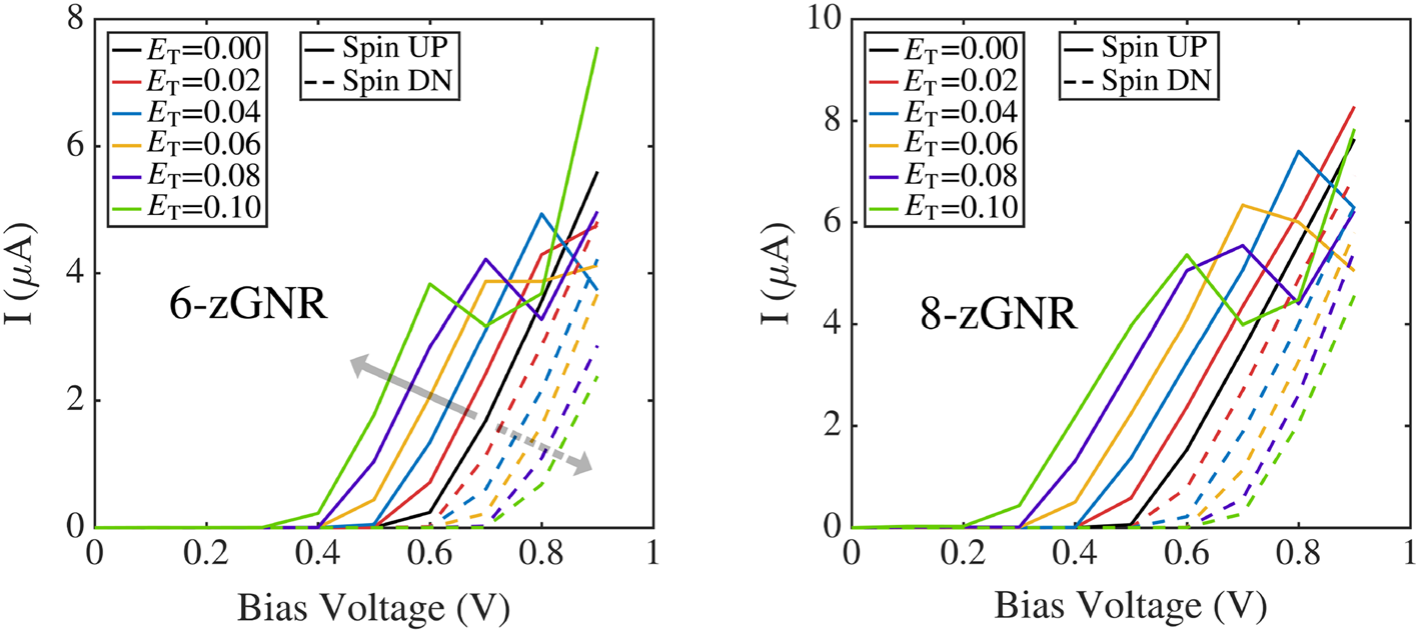
External field effects on the I–V characteristic curves of zGNRs with antiferromagnetic initialization (6-zGNR left, 8-zGNR right). Colors indicate different transverse fields, in units of of ${\mathrm {V/}}\AA$. The solid lines represent spin up current, while the dashed lines represent spin down current. Gray arrows indicate the trends associated with increases in the magnitude of the transverse electric field.

**Figure 3:**
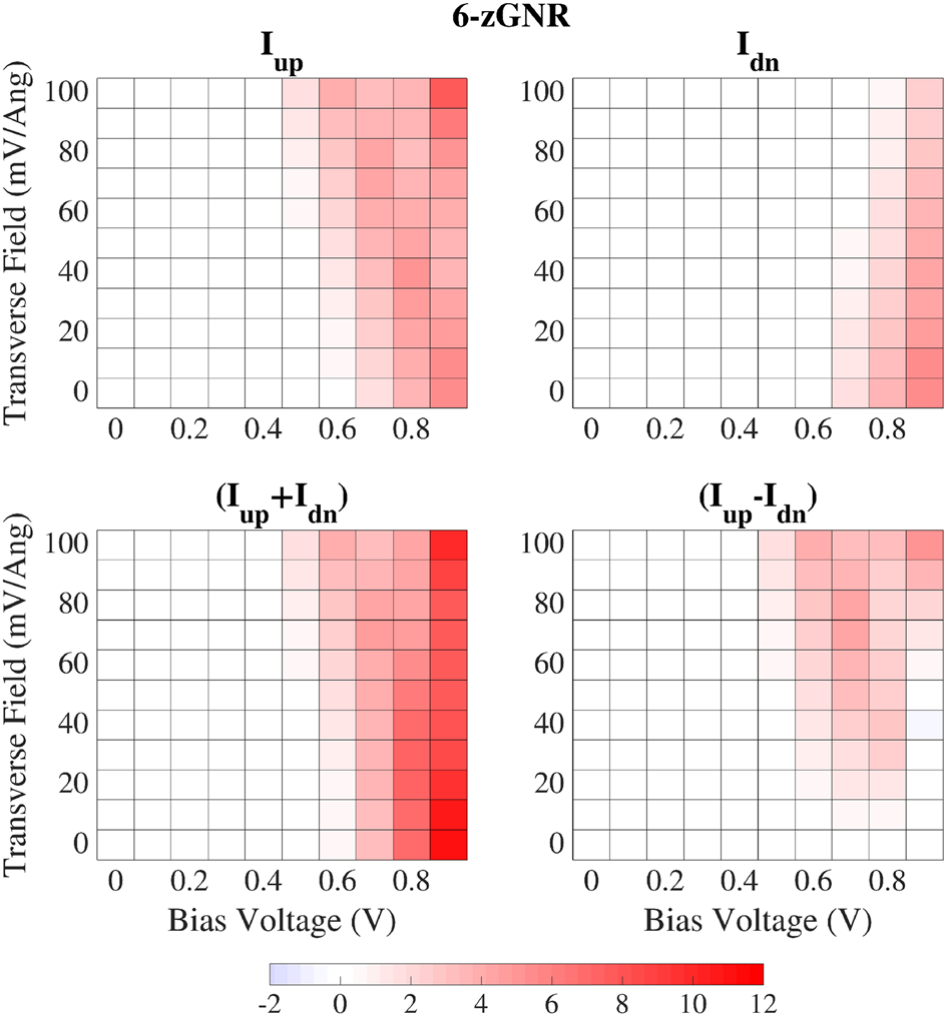
Spin current response of the 6-zGNR as a function of the applied bias voltage and the transverse electric field (current in microamps).

The transmission pathways presented in this paper are computed using the energy averaged local bond transmissions (transmission coefficients between two atoms) over the bias window:

3 $$\begin{aligned} \bar{T}_{ij}=\frac{1}{\left| \mu _{\mathrm {L}} - \mu _{\mathrm {R}} \right| }\displaystyle \int _{\mu _{\mathrm {L}}}^{\mu _{\mathrm {R}}} T_{ij}(E)\,{\mathrm {d}}E \end{aligned}$$

where *i* and *j* indicate two different atoms in the model. This energy averaged bond transmission measures, at zero temperature and at a fixed voltage, the relative magnitudes of the atom-to-atom currents in the nanoribbon. The transmission pathway plots provided in a later section are “normalized with the largest arrow in each plot being the same size, irrespective of the magnitude of the total transmission ...”^43^ and therefore visualize the relative distributions of the spin currents in the zGNR at specified combinations of bias voltage and transverse electric field strength.

## Results and discussion

The characteristic curves shown in Fig. 2 describe the spin current response of antiferromagnetic 6-zigzag and 8-zigzag graphene nanoribbons over the entire range of bias voltages and transverse electric fields considered in this paper. As indicated by the gray arrows shown in Fig. 2a, both the spin up and spin down currents diverge from the zero electric field case (solid black line), more strongly as the magnitude of the transverse field increases. The spin up current traces are non-monotonic, and show in general relative maxima located at bias voltage values which drop as the strength of the applied transverse electric field increases. Comparing Fig. 2 with Fig. 1b, it is: (a) the divergence of the spin up current trace from the zero electric field case, (b) the negative differential resistance shown in the spin up current versus bias voltage curves, and (c) the overarching non-linearity of the zGNR response plots which distinguish the antiferromagnetic results from the ferromagnetic case.

**Figure 4:**
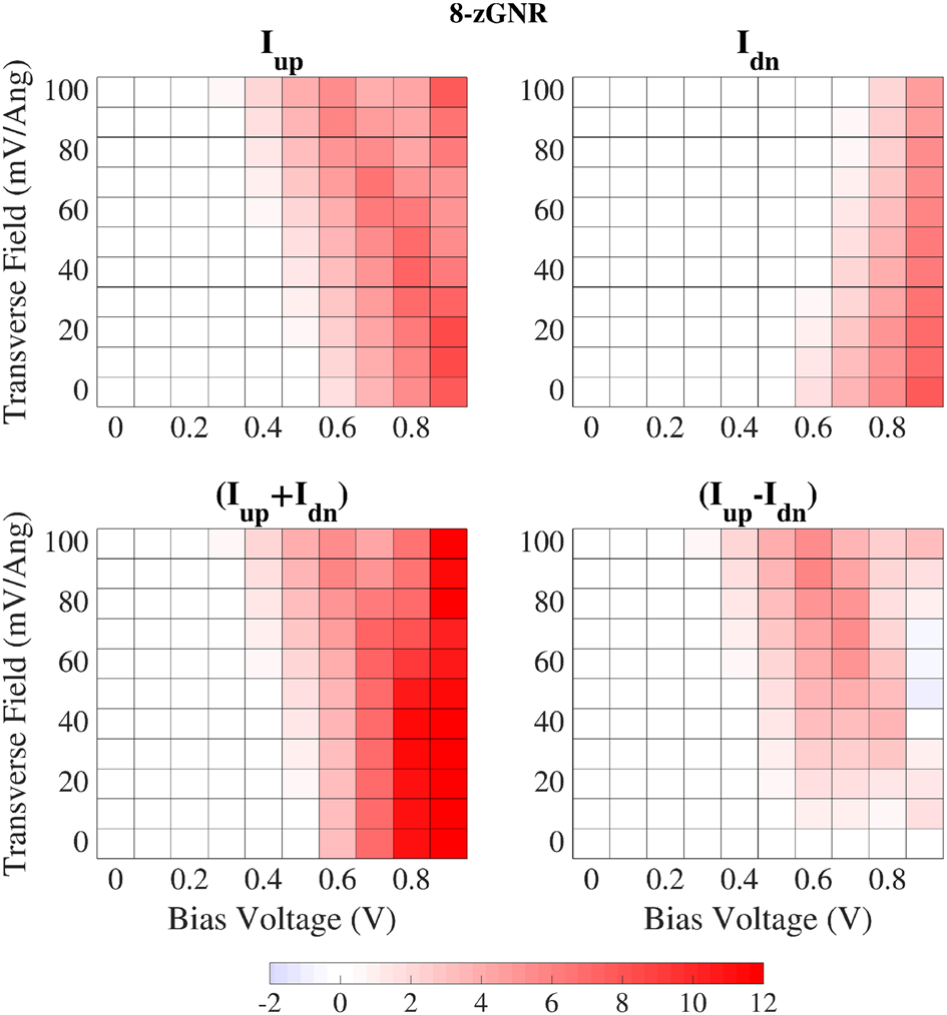
Spin current response of the 8-zGNR as a function of the applied bias voltage and the transverse electric field (current in microamps).

**Figure 5:**
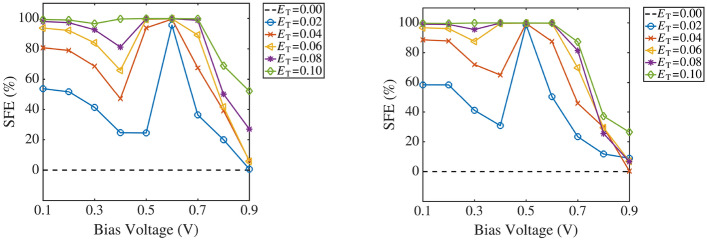
Spin filter efficiencies of the 6-zGNR (left) and the 8-zGNR (right) as a function of the transverse electric field, in units of of ${\mathrm {V/}}\AA$.

Interpretation of the preceding results, from a spin current control perspective, is facilitated by re-plotting the Fig. 2 data, as shown Fig. 3 through 5. Figures 3 and 4 show that adjustment of the operating conditions (bias voltage and electric field strength) at any fixed GNR width can be used to emphasize spin up, spin down, total current, or spin current difference transmission in the conductor. Widening the GNR generally elevates the level of current transmission while maintaining the ability to emphasize particular spin current combinations. Figure 5 plots the spin filtering efficiency (SFE)

4 $$\begin{aligned} {\mathrm {SFE}}= \mid (I_{\mathrm {UP}}-I_{\mathrm {DN}})/(I_{\mathrm {UP}}+I_{\mathrm {DN}})\mid \times 100\% \end{aligned}$$

of the 6-zigzag and 8-zigzag GNR conductors. Note that the bias voltage at which spin filtering efficiency nears 100% for all field strengths is determined by the GNR width. Above that critical bias voltage (to be discussed later), adjustment of the applied transverse field (at any fixed bias) allows for substantial changes in the spin filtering performance over most of the bias voltage range. At bias voltages below the aforementioned critical bias voltage: spin filtering is high at high transverse fields; however, as the transverse field and the bias voltage are both reduced, the plotted SFE is increasingly computed as a ratio of small numbers and should therefore be interpreted with caution. In relating the computed spin current response to the zGNR electronic structure, the paragraphs which follow discuss band diagrams, charge transfer, transmission spectra, and transmission pathways. While the first three metrics are widely used in interpreting quantum conductance physics, the analysis of transmission pathways employs rather recently developed methods^43^. Transmission pathway plots provide valuable insight, since they allow for the direct visualization of scattering physics in very heterogenous electronic structures like the ones considered here.

**Figure 6:**
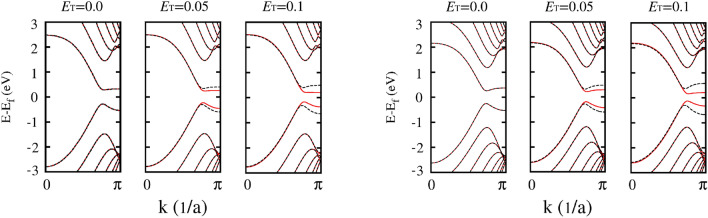
Band diagrams (at zero bias voltage) for the 6-zGNR (left) and the 8-zGNR (right) as a function of the transverse electric field, in units of of ${\mathrm {V/}}\AA$. The solid and dashed lines represent the spin-up and spin-down bands respectively.

Figure 6 shows the effects of the transverse electric field on the (zero bias voltage) band gaps of the 6-zGNR and 8-zGNR conductors, over the full range of transverse field strengths considered in this paper. Note that the maximum transverse electric fields applied in this work are on the order of 25% of those required to fully close the band gap^10,26^ for the spin up state in the modeled zGNRs^4,9^. At all of the field strengths considered here, the band gap of the spin up state is reduced as the applied field is increased, while the while the band gap of the spin down state is enlarged. This band gap response is consistent with the divergent spin current trends indicated by the gray arrows drawn in Fig. 2a, but does not explain the non-monotonic variation of the spin up current shown in Fig. 2a, which appears at higher bias voltage values. Note that the aforementioned critical bias voltages, shown in Fig. 5, at which spin filtering efficiency nears 100% for all field strengths, appear to be determined by the zero bias band gaps for the modeled zGNRs, shown in Fig. 6 (the band gap is reduced as the zGNR width is increased).

**Figure 7:**
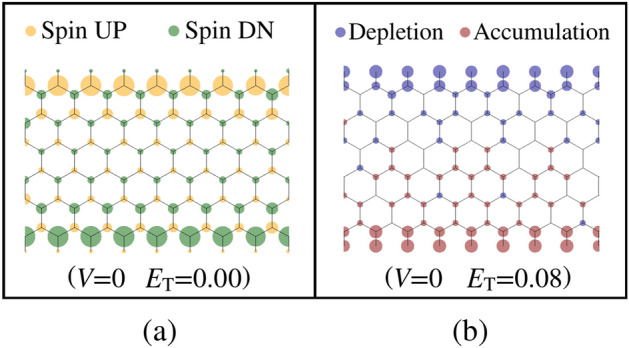
Spin charge distribution for the modeled 6-zGNR under zero bias voltage and zero transverse electric field (left, yellow represents net spin UP and green represents net spin DN). At zero bias, change in the total charge ($Q_{\mathrm {UP}}+Q_{\mathrm {DN}}$) due to the application of a transverse electric field of magnitude $E_{\mathrm {T}}=0.08\,{\mathrm {V/}}\AA$ (right, red indicates charge accumulation and blue indicates charge depletion).

**Figure 8:**
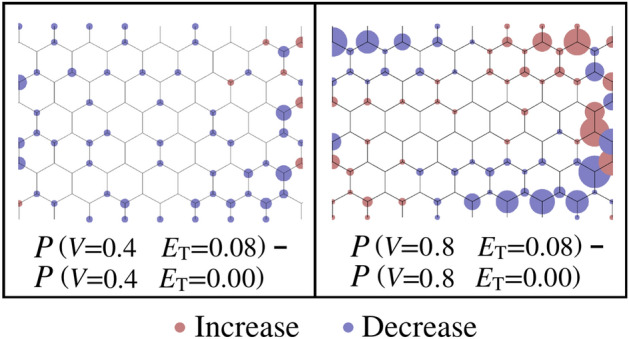
Change in the magnitude of of the spin moment (*P*) due to the application of transverse electric fields of magnitude: $E_{\mathrm {T}}=0.04\,{\mathrm {V/}}\AA$ (left, at a bias voltage of 0.4V) and $E_{\mathrm {T}}=0.08\,{\mathrm {V/}}\AA$ (right, at a bias voltage of 0.8V). Red indicates an increase in the spin moment and blue indicates a decrease in the spin moment.

Mulliken population analysis of the spin charge density distribution in the zGNR provides additional insight relevant to the spin current pathway discussion which follows. In the antiferromagnetic initialized configuration of the zGNR, at zero bias, the spin distribution (Fig. 7a) is antisymmetric about the GNR longitudinal axis, with no longitudinal space dependence. Upon application of the transverse field (Fig. 7b), the total charge ($Q_{\mathrm {UP}}+Q_{\mathrm {DN}}$, where *Q* denotes charge) undergoes a longitudinally uniform change which is antisymmetric about the longitudinal zGNR axis (charge is depleted along one edge and accumulated along the opposite edge). Under the subsequent application of a bias voltage, the longitudinal antisymmetry of the charge distribution is disturbed, and both the longitudinal and transverse spatial charge gradients in the zGNR are accentuated as the bias voltage is increased. Figure 8 depicts the change in the magnitude of the spin moment (P), defined as

5 $$\begin{aligned} P = \left| Q_{\mathrm {UP}}-Q_{\mathrm {DN}} \right| \end{aligned}$$

due to a change in the bias voltage at the maximum transverse field strength considered in this paper. The heterogeneous nature of the charge distribution illustrated in Fig. 8 is consistent with the development of spatially complex transmission pathways, observed in the atom-to-atom transmission pathway plots which follow.

**Figure 9:**
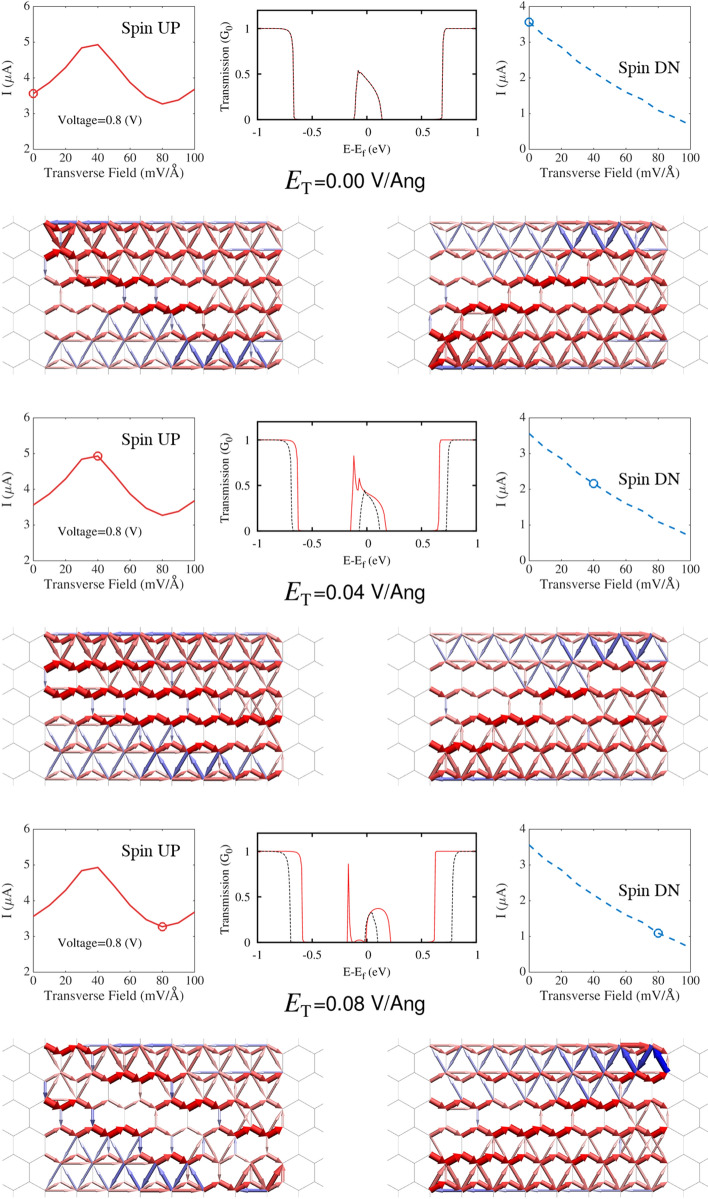
Spin currents, transmission spectra, and transmission pathways for a 6-zGNR at a bias voltage of $0.8\,{\mathrm {V}}$ with transverse electric fields of magnitude 0.0 ${\mathrm {V/}}\AA$ (top), 0.04 ${\mathrm {V/}}\AA$ (middle), and 0.08 ${\mathrm {V/}}\AA$ (bottom).

The most striking feature of the antiferromagnetic zGNR I–V curves presented in Fig. 2 is the non-monotonic variation of the spin up current with bias voltage. Figure 9 shows the variation of spin currents, transmission spectra, and transmission pathways in a 6-zGNR, at a bias voltage of 0.8 volts, for three different values of the applied transverse electric field strength. These plots (and the preceding bad gap analysis) suggest the cause-effect physics responsible for the non-monotonic traces of the spin up curves as well as the more ordinary traces of the spin down curves: (a) At zero transverse field, the spin up and spin down currents are equal, the spin up and spin down transmission spectra overlap, and the transmission pathways for the spin up and spin down currents are approximately mirror images (reflection of the spin up pathway plot across the nanoribbon centerline matches the spin down pathway plot). (b) As the transverse field is increased to $0.04\,{\mathrm {V/}}\AA$, the spin up band gap drops and the spin down band gap increases, while the transmission spectra diverge. The transmission pathways remain highly polarized (spin up dominated on the upper half of the GNR, spin down dominated on the lower half of the GNR), however the spin up current concentration moves toward the nanoribbon centerline. At this field strength, the most important effects of the transverse field are to change the band gaps and concentrate the spin up current; the spin up current reaches a relative maximum. (c) At the highest transverse field strength ($0.08\,{\mathrm {V/}}\AA$), the most important effect of the applied field is to disrupt the axial flow path of the majority (spin up) current, scattering the electrons against the GNR edge. The spin up current pathway is now split, and the results suggest the development of spatially periodic spin current transmission structures in the modeled zGNR. Note that at the highest modeled field strength shown in Fig. 9 the spin down flow path, focused on the lower half of the GNR, is little affected and the increase in the spin down band gap further reduces the spin down current flow.

Unlike previous work, the transmission pathways depicted in Fig. 9 are neither inferred from charge distributions or other indirect measures, or restricted to particular energy levels. Hence they offer a distributed property description of the overall current transmission, of particular value in device design or the analysis of cause-effect physics in a heterogeneous system. The Supporting Information provides AVI animations depicting the evolution of the spin up and spin down transmission pathways in 6-zGNR and 8-zGNR over the entire range of transverse field strengths modeled in this paper. It also includes additional analyses performed at higher transverse field strengths and for longer GNRs.

## Conclusion

The I–V characteristics of antiferromagnetic zGNRs under transverse electric fields have received relatively limited research attention, reflecting doubts regarding their magnetic order and energy stability, as well as the feasibility of precise fabrication of narrow zGNRs. Recent experimental and computational work strongly supports the proposition that the precise fabrication of very narrow zGNRs is feasible, and that they will be energetically stable in an antiferromagnetic configuration. Ab initio calculations indicate that the highly nonlinear response of such zGNRs under combined bias voltage and transverse electric fields offers opportunities for spin current control which move beyond conventional spin filtering. The principal effects of the transverse field are to change the zGNR band gaps and to modify the transmission pathways in the zGNR conductor. As illustrated in the present work, the relatively new analysis methods developed to describe transmission pathways in semiconducting nanowires can complement widely used band gap, transmission spectra, and charge transfer analysis to assist in the understanding of spin current transmission in zGNR based spintronic applications.

## Supplementary Information

Supplementary Information.

Supplementary Information.

Supplementary Information.

## Data Availability

The authors contributed equally to this work.
