# Supplementary material for: Spin current distribution in antiferromagnetic zigzag graphene nanoribbons under transverse electric fields
Source: Sci Rep. 2021 Aug 24;11:17088. doi: 10.1038/s41598-021-96636-6 (PMC8385052; doi:10.1038/s41598-021-96636-6)
Supplement: Supplementary file 1 — Supplementary Information. [file 41598_2021_96636_MOESM1_ESM.pdf]

SUPPLEMENTARY MATERIAL

Spin current distribution in antiferromagnetic zigzag graphene nanoribbons under transverse electric fields

Jie Zhang and Eric P. Fahrenthold*

Department of Mechanical Engineering, University of Texas, Austin, TX 78712

* E-mail: epfahren@mail.utexas.edu

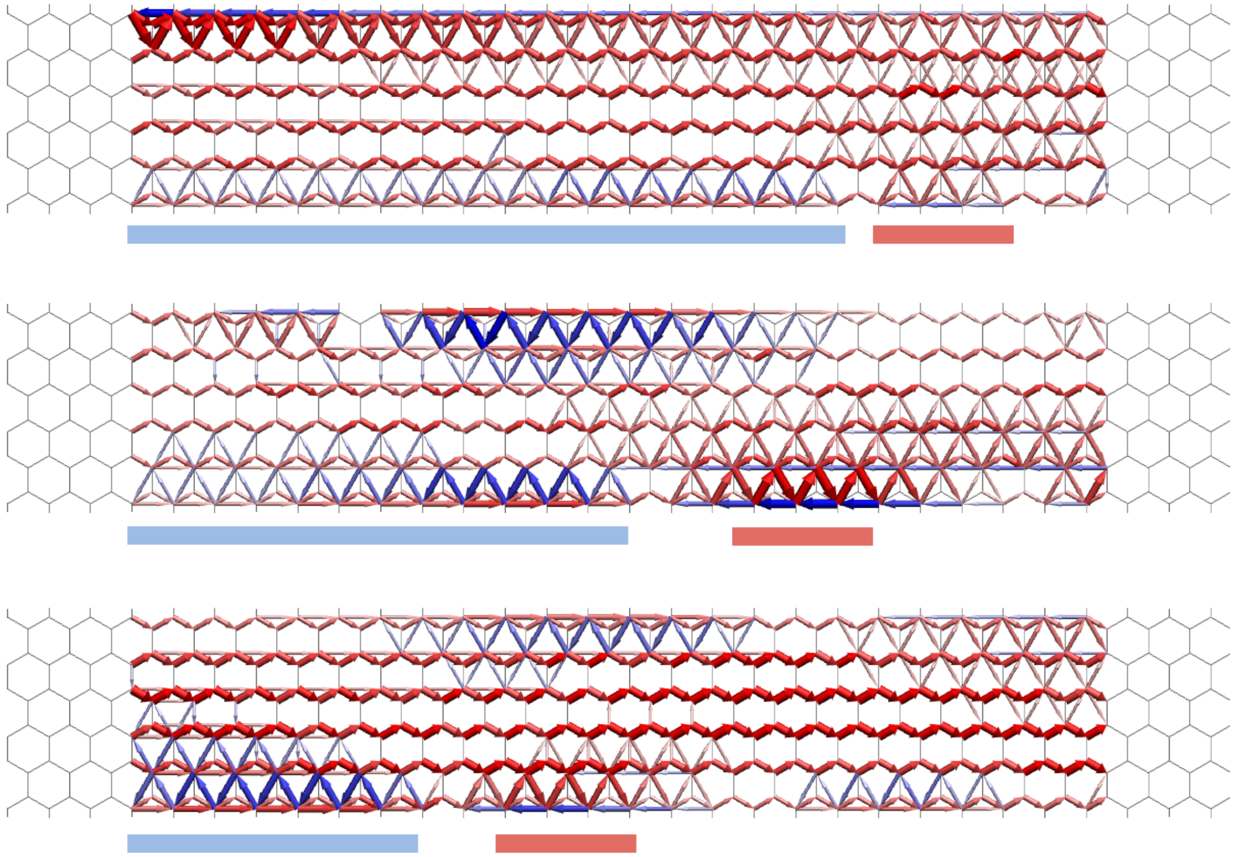

Figure S1: Transmission pathways for the spin up current in a 6-zGNR with a length of 30 unit cells, at a bias voltage of 0.5 V. The applied transverse electric fields are (from top to bottom): 0.00, 0.25, and 0.50 V/Å. The red and blue bars below each pathway plot delineate transmission dominated and reflection dominated regions near the zGNR edges. These results for long nanoribbons and high field strengths suggest that the edge reflections discussed in the paper form periodic structures and that the period is a function of the transverse field strength.

Attached file (SupplementaryVideo1.avi): depicts the evolution of the transmission pathways in 6-zGNR over the entire range of transverse field strengths modeled in this paper.

Attached file (SupplementaryVideo2.avi): depicts the evolution of the transmission pathways in 8-zGNR over the entire range of transverse field strengths modeled in this paper.
